# Supplementary material for: A Multi-Approach for In Silico Detection of Chromosome Inversions in Mosquito Vectors
Source: Microorganisms. 2025 Sep 24;13(10):2231. doi: 10.3390/microorganisms13102231 (PMC12565792; doi:10.3390/microorganisms13102231)
Supplement: Supplementary file 1 [file microorganisms-13-02231-s001.zip › microorganisms-3825310-supplementary.pdf]

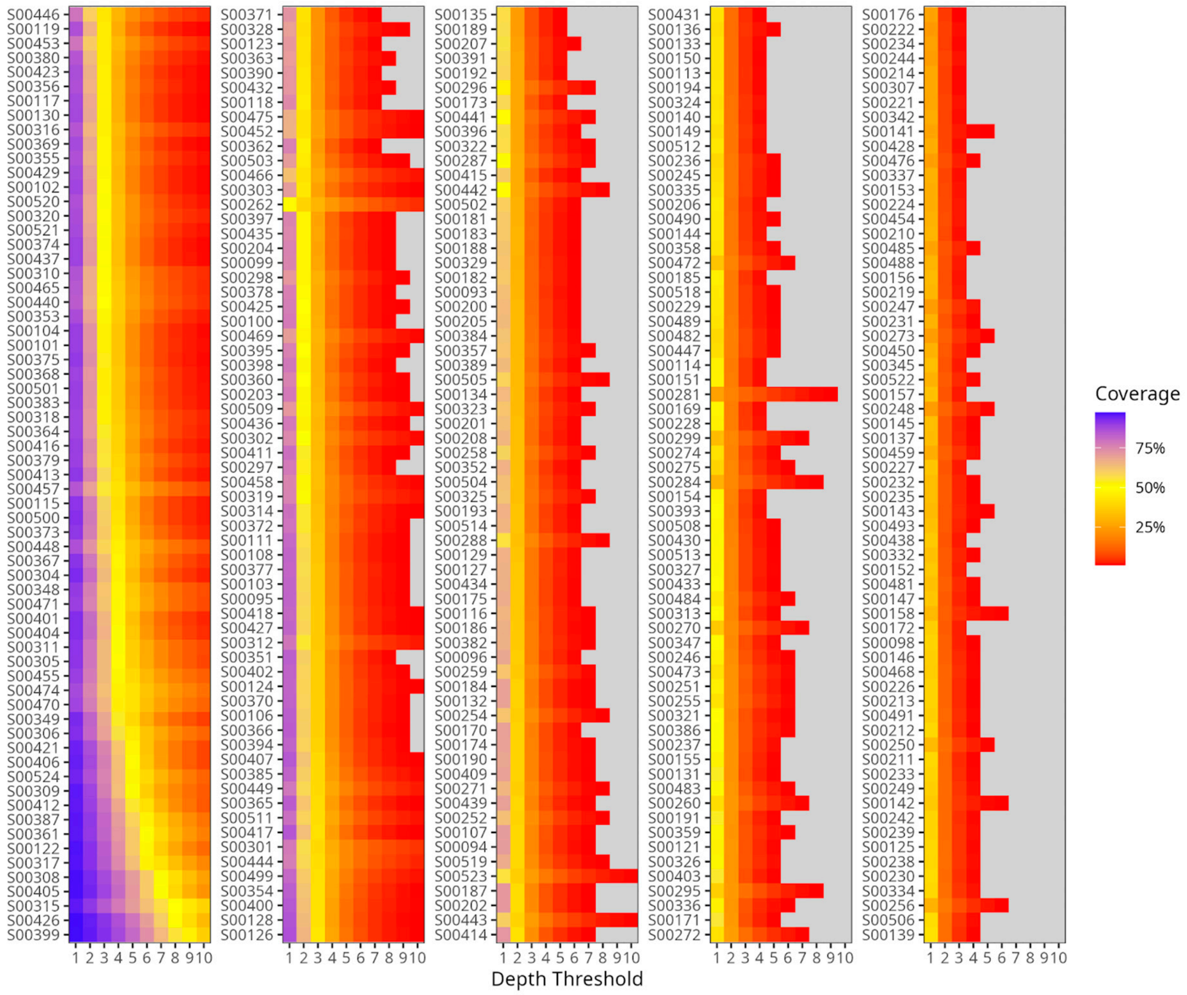

Figure S1. Heatmap of sequencing coverage across samples. Y-axis represents individual sample IDs. X-axis indicates the sequencing depth level. Color scale corresponds to the proportion of the genome covered at the respective depth level. Grey indicates zero coverage.
